# MiR-101 targets DUSP1 to regulate the TGF-β secretion in sorafenib inhibits macrophage-induced growth of hepatocarcinoma

**DOI:** 10.18632/oncotarget.4089

**Published:** 2015-05-29

**Authors:** Xufu Wei, Chengyong Tang, Xiuxian Lu, Rui Liu, Mi Zhou, Diao He, Daofeng Zheng, Chao Sun, Zhongjun Wu

**Affiliations:** ^1^ Department of Hepatobiliary Surgery, The First Affiliated Hospital of Chongqing Medical University, Chongqing, China; ^2^ Department of Pathology and Division of Biological Sciences, University of California San Diego, La Jolla, California, USA; ^3^ Department of Clinical Pharmacology, The First Affiliated Hospital of Chongqing Medical University, Chongqing, China

**Keywords:** hepatocellular carcinoma, miR-101, macrophage, DUSP1, sorafenib

## Abstract

Hepatocellular carcinoma (HCC)-associated macrophages accelerate tumor progression via growth factor release. Therefore, tumor-associated macrophages (TAMs)-initiated signaling cascades are potential therapeutic targets. To better understand anticancer effects of systemic HCC therapy, we studied sorafenib's effect on macrophage function, focusing on macrophage-related growth factor secretion. We found that dual specificity phosphatase 1 (DUSP1) is a direct target of miR-101. Transfection of miR-101 reduced DUSP1 induction in M2 macrophages and prolonged ERK1/2, p38 and JNK activation, whereas inhibition of miR-101 enhanced DUSP1 expression and decreased ERK1/2, p38 and JNK activation. miR-101 expression was decreased by sorafenib, and inhibition of PI3K/AKT blocked induction of miR-101 by LPS in M2 cells. M2 cells with greater TGF-β and CD206 mRNA expression compared to M1 cells had increased hepatoma growth, metastases and EMT. Sorafenib inhibited miR-101 expression and enhanced DUSP1 expression and lowered TGF-β and CD206 release in M2 cells, slowing macrophage-driven HCC. Our studies demonstrate miR-101 regulates macrophage innate immune responses to LPS via targeting DUSP1. Sorafenib alters macrophage polarization, reduces TGF-β driven cancer growth, metastases and EMT *in vitro*, and partially inhibits macrophage activation *in vivo*. Thus, macrophage modulation might explain the anticancer effects of sorafenib.

## INTRODUCTION

Nearly 500,000 people are diagnosed with hepatocellular carcinoma (HCC) each year, and their overall 5-year survival rate is less than 12% [1]. Advanced liver cirrhosis and impaired liver function are also critical components of the tumor microenvironment (TME), but tumor-associated macrophages (TAMs) in the tumor stroma can affect tumor prognosis via participation in innate and adaptive immunity [2].

TAMs are an infiltrating macrophage subpopulation derived from circulating monocytes with a M2 phenotype activated by type 2 T helper cells (Th2) cytokines, such as interleukin-4 (IL-4) [3]. TME is key to directing macrophages to express the M2 phenotype and promotes cytokines secretion into the TME to enhance tumorigenesis and metastases [4]. Abundant TAMs correlate with poor prognosis of solid tumors, including liver cancer [5]. TAMs typically resemble a M2-polarization within the functional spectrum of macrophages, which transmits a series of signaling cascades leading to mitogen-activated protein kinases (MAPKs) activation [6]. MAPKs are highly conserved serine/threonine protein kinases that include ERKs, JNK/stress-activated protein kinase and p38 MAPKs. Once activated, MAPKs phosphorylate downstream protein kinases and transcription factors, leading to the production of proinflammatory cytokines, such as TNF-α, IL-1, IL-6, and endothelial growth factor (EGF). These inflammatory mediators facilitate tumor angiogenesis, extracellular matrix degradation and remodeling, and promote tumor cell motility [7].

Deactivation of MAPKs is regulated mainly by a family of MAPK phosphatases [8]. Originally identified as an immediate early gene, DUSP1 later confirmed to be a dual-specificity phosphatase and a negative regulator of MAPKs activities [9–12]. DUSP1-deficient macrophage cells (M2) have prolonged p38 and JNK/stress-activated protein kinase activation and enhanced production of TNF-α and IL-6 compared with wild-type cells [13]. Moreover, DUSP1 knockout mice produce substantially greater quantities of inflammatory cytokines and have greater mortality from endotoxic shock [14].

miRNAs are small, highly conserved noncoding RNAs known to suppress expression of protein-coding genes through imperfect complementarity with the 3′-untranslated region (3′-UTR) of target mRNA [15]. Previously, we reported that miR-101 expression is decreased in HCC tissues and hepatoma cells [16]. Recently, miRNAs have been shown to be involved in innate immune responses; specifically, in response to stimulation by LPS or other microbial components, a rapid increase in selected miRNA expression occurs and has been observed in monocytic cell lines or mouse macrophages [17, 18].

Sorafenib is a small molecule multi-kinase inhibitor approved for systemic HCC treatment and has become the standard therapy for patients with advanced HCC. How sorafenib works is unclear, so we investigated the drug's mechanism of action on macrophage-induced tumor development.

In this study, we investigated the influence of sorafenib on macrophage polarization and macrophage-dependent secretion *in vitro*, and we explored macrophage and tumor cell interactions in HCC patients. DUSP1 was identified to be a target of miR-101, a tumor-related miRNA that is induced after cellular activation via LPS. Induction of miR-101 by LPS depends on the PI3K/AKT pathway. We also observed that miR-101 regulates sorafenib-induced production of DUSP1 and subsequent MAPKs activation in macrophages. We thus suggest the targeting of DUSP1 by miR-101 regulates MAPKs activation during sorafenib-mediated inhibition of macrophage-induced hepatocarcinoma growth.

## RESULTS

### Phenotype and function of polarized monocyte-derived macrophage cultures

**Figure 1:**
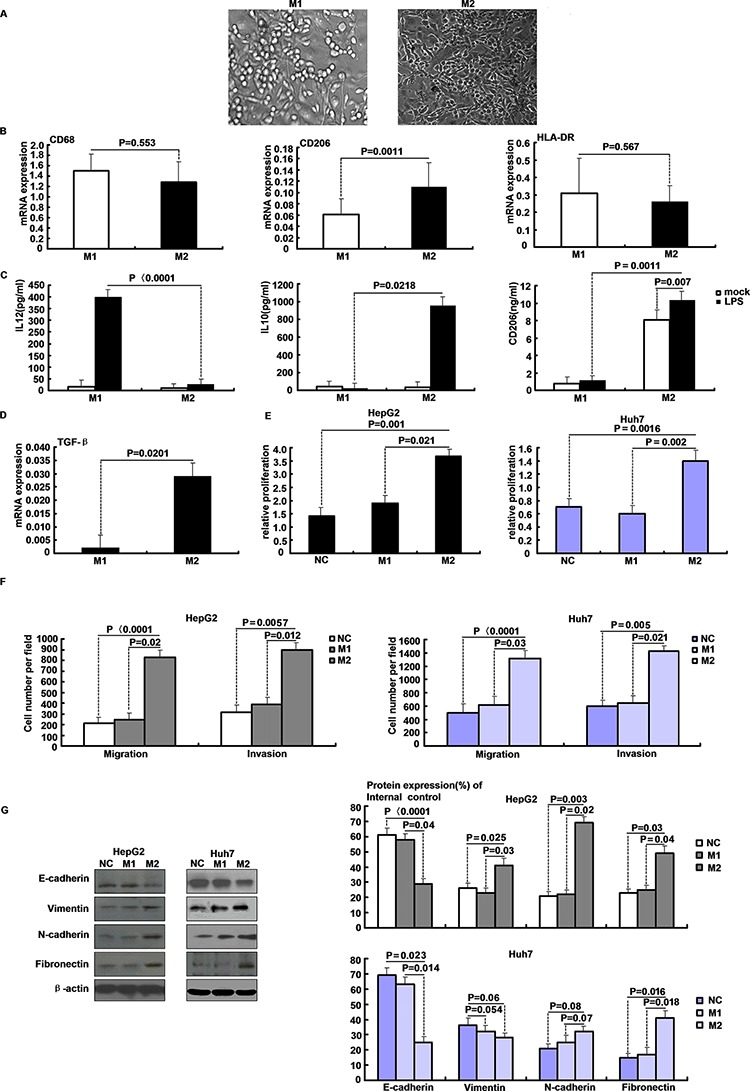
Features of M1 and M2 polarized macrophages in culture **A.** Phase contrast microscopy of M1 and M2 macrophages cultures after one week in presence of GM-CSF (800 IU/ml) or M-CSF (100ng/ml), respectively. **B.** mRNA expression of CD68, CD206 and HLA-DR in M1/M2 macrophages quantified by qRT-PCR. **C.** IL-12, IL-10 and CD206 concentrations in cell culture medium of M1/M2 macrophages after LPS stimulation for 3 h were determined by ELISA. **D.** TGF-β expression in M1/M2 macrophages was determined by qRT-PCR. **E.** Proliferation of HepG2 and Huh7 cells in the presence of M1/M2 macrophages conditioned media. **F.** Migratory and invasive abilities of HepG2 and Huh7 cells in macrophages supernatant as evaluated by the transwell assay. **G.** Western blot analysis of EMT-related proteins levels after M2 supernatants transfected in HepG2 and Huh7 cells.

Monocyte-derived M1 and M2 macrophage cultures were established to study proliferation of hepatoma cell lines in the presence of growth factors originating from macrophages. Cultured M1 macrophages developed classical round macrophage morphology in contrast to the bipolar appearance of cultured M2 macrophages (Figure 1A). CD68 and HLA-DR mRNA expression confirmed macrophage polarization and differentiation in both cell types, whereas differential expression of CD206 indicated an alternative polarization state in M2 macrophages (Figure 1B). Functional polarization into M1 and M2 macrophages resulted in IL-12 or IL-10 dominated cytokine secretion upon LPS stimulation, respectively. Shedding of CD206 by M2 macrophages further confirmed their alternative polarization (Figure 1C). Transforming growth factor beta (TGF-β) expression was measured in both polarization states and was markedly increased in M2 cultures (Figure 1D). Proliferation assays in the presence of macrophage-conditioned culture media were performed to verify functional relevance of secreted growth factors for hepatoma cell growth. HepG2 and Huh7 cells proliferated faster in the presence of M2 compared to M1 macrophage conditioned cell culture media or unconditioned controls (Figure 1E). Cell motility was quantified in both cell lines using a Boyden chamber assay. M2 secreting supernatants had greater motility relative to M1 and NC in HepG2 and Huh7 cells (Figure 1F). To investigate invasiveness of the cells, an *in vitro* invasion assay was performed. M2 had more invading cells compared with M1 and NC (Figure 1F). Epithelial to mesenchymal transition (EMT) is a crucial step in cancer cell metastasis. As shown in Figure 1G, M2 supernatants transfected in HepG2 and Huh7 cells down-regulated E-cadherin expression and up-regulated vimentin, N-cadherin, and fibronectin expression. Thus M1/M2 macrophages are suitable for studying hepatoma progression in response to macrophage-derived growth factors.

### miR-101 represses DUSP1 expression through 3′-UTR interactions

**Figure 2:**
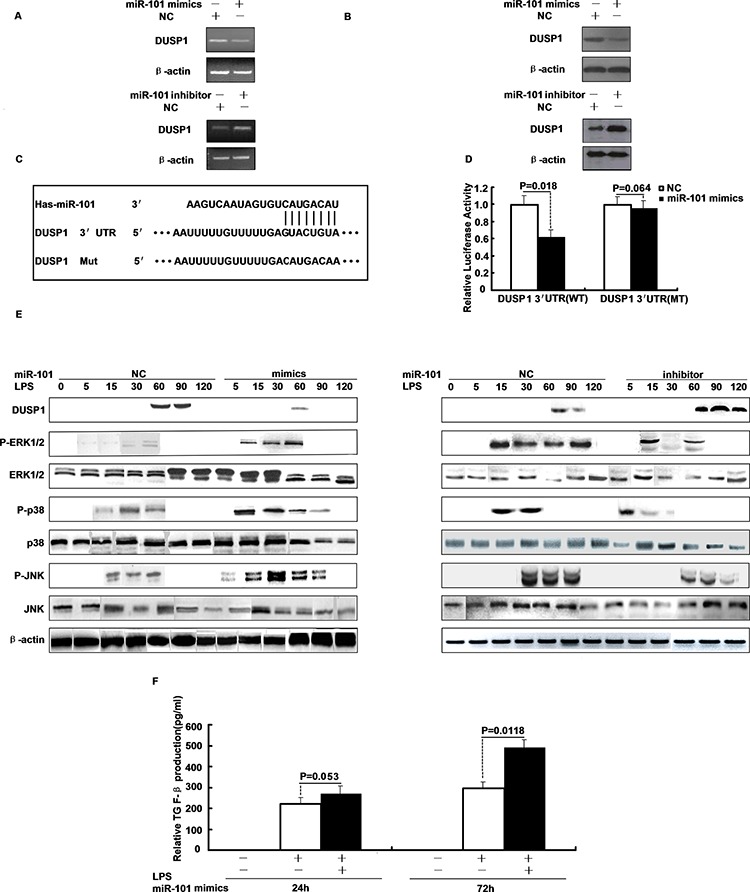
DUSP1 is a direct target of miR-101 and miR-101 regulates the LPS-induced activation of p38 and JNK **A.** The mRNA and **B.** protein levels of DUSP1 were determined in M2 cells were transfected with miR-101 mimics, inhibitor or respective controls. **C.** The putative human DUSP1 3′-UTR fragment containing wild-type or mutant miR-101-binding sequence was inserted into a plasmid downstream of the luciferase reporter gene. **D.** Dual-luciferase assay of 293T cells co-transfected with the firefly luciferase constructs containing the DUSP1 WT or Mut 3′-UTR as well as miR-101 mimics or negative control. **E.** M2 cells were transfected with miR-101 mimics, inhibitor or respective controls and then stimulated with LPS for different time periods, Western blot analysis of DUSP1, ERK1/2, p38 and JNK protein expression. **F.** ELISA analysis of TGF-β level after M2 cells were transfected with miR-101 mimics or NC and then stimulated with LPS.

It is accepted generally that miRNAs exert their function through regulation of downstream target gene expression. To examine the effect of miR-101 on DUSP1 expression, M1 and M2 macrophage cells were transfected with miR-101 mimics or negative control and stimulated with LPS. We found that over-expression of miR-101 caused a significant decrease in DUSP1 mRNA (as measured by qRT-PCR) and protein level (as measured by Western blot) in M2 cells (Figure 2A–2B). Next, we transfected the miR-101 inhibitor or negative control into M1 and M2 macrophage cells and stimulated with LPS, the results showed that inhibition of endogenous miR-101 expression resulted in up-regulation of DUSP1 mRNA and protein level in M2 cells (Figure 2A–2B), but M1 is not different (data not shown). Thus, to determine putative miR-101 targets, we performed target prediction analysis using TargetScan and miRanda prediction programs. To validate whether DUSP1 is a bona fide target of miR-101, a human DUSP1 3′-UTR fragment containing wild-type or mutant miR-101-binding sequence (Figure 2C) was inserted into a plasmid downstream of a firefly luciferase reporter gene, and the plasmids were transfected into 293T cells. As predicted, the relative luciferase activity of the wild-type 3′-UTR reporter was significantly suppressed in cells co-transfected with miR-101 mimics compared to cells co-transfected with control RNA (Figure 2D). In contrast, the luciferase activity from the mutant 3′-UTR reporter was no longer able to be suppressed by miR-101 mimics in cells co-transfected with miR-101 mimics compared to cells co-transfected with control RNA (Figure 2D). These results indicate that miR-101 functions to directly suppress DUSP1 gene expression through the miR-101-binding sequence located at the 3′-UTR of the DUSP1 mRNA.

### miR-101 regulates the LPS-induced activation of ERK1/2, p38 and JNK and induces production of TGF-β

Because miR-101 negatively regulates the expression of DUSP1, which is known as a phosphatase of MAPKs, we next examined whether miR-101 plays a role in the activation of MAPKs. M2 macrophage cells were transfected with miR-101 mimics or miR-101 inhibitor and then stimulated with LPS. Activation of MAPKs was measured by Western blot. LPS stimulation induced the expression of DUSP1 protein which peaked at 60 min, but transfection with miR-101 mimics markedly inhibited DUSP1 expression and prolonged the phosphorylation of ERK1/2, p38 and JNK apparently to 60, 90 or 120 min (Figure 2E). In contrast, treatment of cells with miR-101 inhibitor enhanced the expression of DUSP1 and attenuated the phosphorylation of ERK1/2, p38 and JNK (Figure 2E). These results suggest that miR-101 may regulate the LPS-induced activation of ERK1/2, p38 or JNK through targeting DUSP1. Because miR-101 has been shown to regulate the expression of DUSP1 and subsequent activation of MAPKs, we then examined whether miR-101 regulates TGF-β production. M1 and M2 cells were transfected with miR-101 mimics and then stimulated with LPS and TGF-β was measured with ELISA. Figure 2F shows that miR-101 mimics increased TGF-β up to 1.7-fold in M2 cells compared with control treatment, and M1 cells were not different (data not shown).

### The PI3K/AKT pathway regulates the induction of miR-101 by LPS

**Figure 3:**
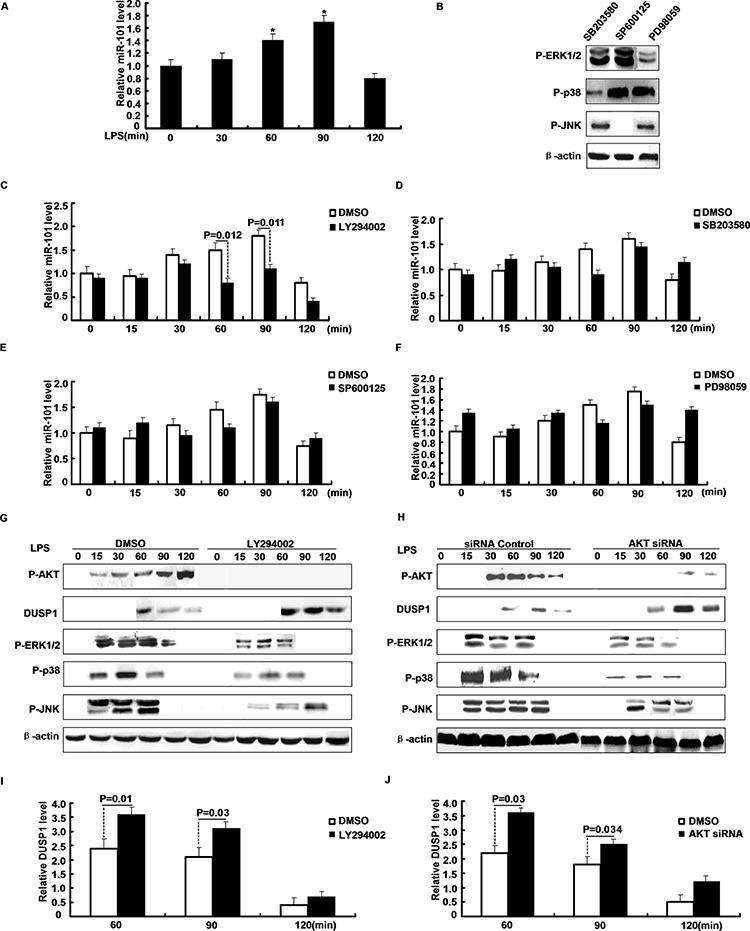
PI3K/AKT pathway regulates the induction of miR-101 by LPS **A.** M2 cells were stimulated with LPS and miR-101 expression was determined by qRT-PCR. **B.** Western blot analysis of protein level after M2 cells were pretreated with inhibitors and then stimulated with LPS. **C–F.** miR-101 mRNA levels were determined after M2 cells were pretreated with (C) LY294002, (D) SB203580, (E) SP600125, or (F) PD98059 and then stimulated with LPS for different time periods as indicated. **G.** M2 cells were pretreated with PI3K/AKT inhibitor, **H.** transfected with AKT siRNA or NC and then stimulated with LPS for the indicated time periods, Western blot analysis of DUSP1, ERK1/2, p38, JNK protein expression. **I–J.** Densitometric analysis of DUSP1 protein expression as G or H.

Stimulation by LPS triggers the activation of multiple signaling pathways. Treatment of M2 cells with LPS enhanced miR-101 expression in a time-dependent manner that reached a peak in ~90 min (Figure 3A). We next analyzed pathways regulated after LPS-stimulated induction of miR-101 using different kinase inhibitors. PI3K/AKT inhibitor LY294002, ERK inhibitor PD98059, p38 inhibitor SB203580 or JNK inhibitor SP600125 specifically inhibited corresponding kinase. M2 cells were pretreated with inhibitors and then stimulated with LPS and miR-101 expression was measured with qRT-PCR. Only inhibition of PI3K/AKT by LY294002 markedly attenuated LPS-induced expression of miR-101. Treatment with SB203580, SP600125, or PD98059 did not have a significant effect on miR-101 induction by LPS (Figure 3B–3F). Furthermore, upon LPS stimulation, treatment with LY294002 increased production of DUSP1 and shortened activation of ERK1/2, p38 and JNK (Figure 3G, 3I). Specific inhibition of AKT by siRNA also gave similar results (Figure 3H, 3J). Thus, part of the effect of miR-101 may occur through inhibition of LPS-induced activation of PI3K/AKT, thus reducing expression of miR-101 and subsequently inducing DUSP1 to deactivate MAPKs.

### Sorafenib inhibits miR-101 expression and inhibits growth factor expression in macrophages

**Figure 4:**
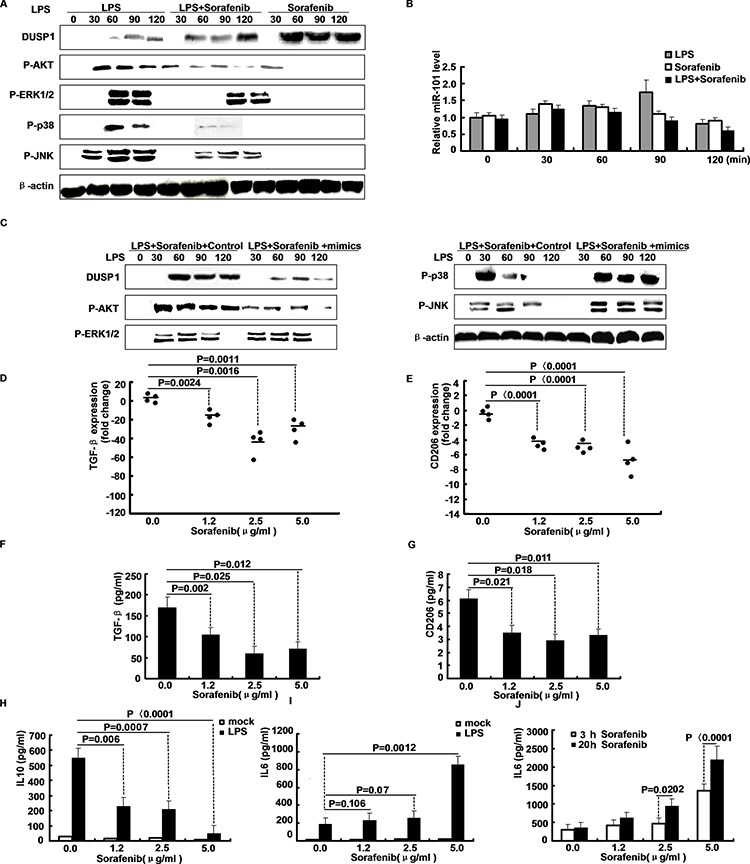
Sorafenib inhibits LPS-induced miR-101 expression and affects growth factor expression in macrophages **A.** M2 cells were treated with LPS, LPS plus Sorafenib, or Sorafenib only for different time periods, Western blot analysis of DUSP1, ERK1/2, p38, JNK protein expression. **B.** M2 cells were treated as indicated, expression of miR-101 was analyzed by qRT-PCR. **C.** M2 cells were treated with LPS plus sorafenib together with miR-101 mimics or NC for different time points, Western blot analysis of DUSP1, AKT, ERK1/2, p38, JNK protein expression. **D–E.** TGF-β and CD206 mRNA expression by qRT-PCR after M2 macrophages were treated with sorafenib for 24 h at indicated concentrations. **F–G.** TGF-β secretion and CD206 were confirmed by ELISA. **H–I.** IL-10 and IL-6 levels in sorafenib-pretreated M2 macrophage cultures were determined by ELISA following stimulation with LPS for 3 h as indicated. **J.** IL-6 induction was assessed by ELISA in a sorafenib time-course experiment after 3 h and 20 h of treatment, following stimulation with LPS for additional 3 h.

Sorafenib is one of the most effective anti-cancer drugs that inhibit activation of MAPKs. To examine whether sorafenib alters miR-101, M2 cells were treated with LPS, LPS plus sorafenib, or sorafenib only for different time periods. After LPS stimulation, treatment sorafenib markedly increased DUSP1 expression and attenuated activation of ERK1/2, p38 and JNK (Figure 4A). Also, treatment with sorafenib inhibited LPS-stimulated activation of PI3K/AKT and the LPS-induced expression of miR-101(Figure 4B). So, up-regulation of DUSP1 by sorafenib may correlate with down-regulation of miR-101 due to AKT inhibition. We then measured the effect of miR-101 mimics on the up-regulation of DUSP1 by sorafenib in LPS-stimulated M2 cells. Figure 4C indicates that treatment with miR-101 mimics and sorafenib inhibited induction of DUSP1 in LPS-stimulated M2 cells. Therefore, part of the anti-inflammatory effect of sorafenib may occur through inhibition of LPS-induced activation of PI3K/AKT, reducing miR-101 expression and subsequent induction of DUSP1 to deactivate MAPKs. Treatment of M2 macrophages with increasing sorafenib concentrations decreased TGF-β and CD206 mRNA expression (Figure 4D–4E). In line with mRNA expression data, TGF-β and CD206 protein was suppressed in M2 macrophage culture-derived media by sorafenib (Figure 4F–4G). The decrease in CD206 also indicated a reversion of alternative macrophage polarization, particularly because LPS-stimulated IL-10 secretion (Figure 4H) by M2 macrophages was reduced in favor of a classical IL-6 response after addition of sorafenib in a dose-and time-dependent fashion (Figure 4I–4J). However, M1 macrophages did undergo cytokine induction and macrophage morphology did not change during sorafenib treatment.

### Sorafenib inhibits HCC progression through TGF-β

**Figure 5:**
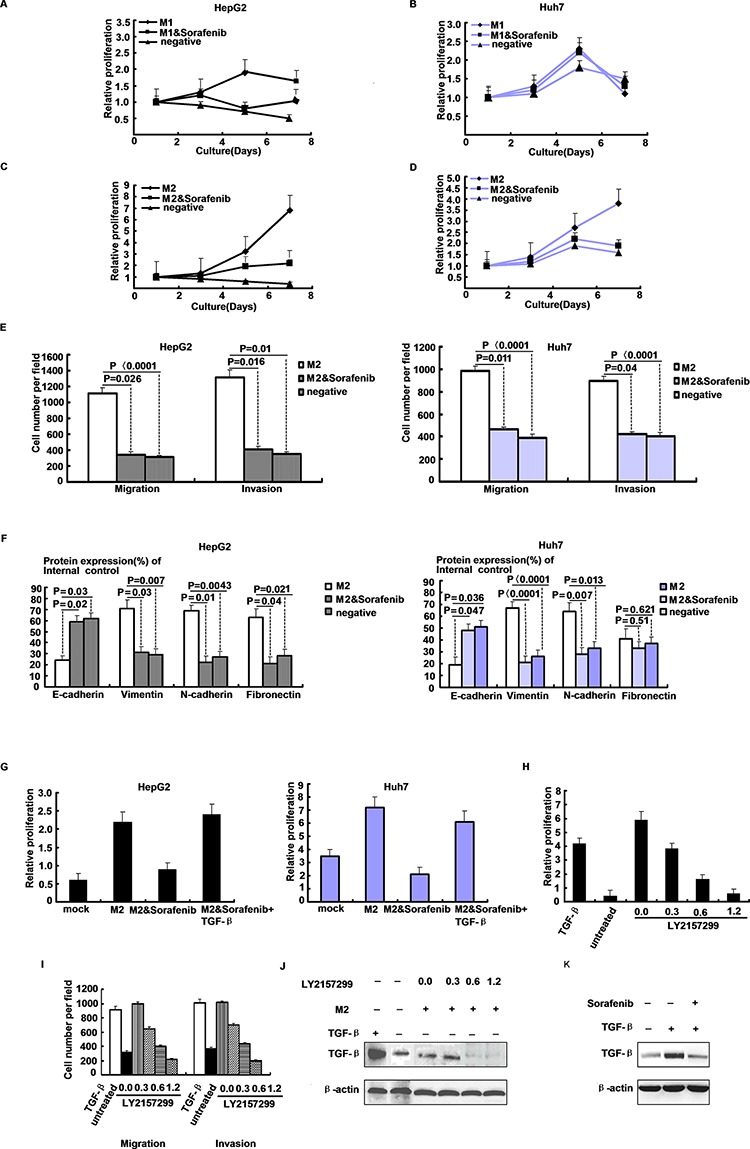
Macrophage secreted TGF-β drives HCC progression **A–D.** Proliferation of HepG2 and Huh7 cells in presence of conditioned media derived from sorafenib-treated (5 ug/ml, 24 h) or treated M1/M2 macrophages by CCK-8 assay. **E.** Migratory and invasive abilities of HepG2 and Huh7 cells evaluated by the transwell assay after treatment with sorafenib in M2 supernatant. **F.** Densitometric analysis of EMT-related proteins levels after HepG2 and Huh7 cells treated with M2 supernatant and sorafenib. **G.** HepG2 and Huh7 cells growth after supplementation of TGF-β (100 ng/ml) to media derived from M2 macrophage cultures treated with sorafenib (5 ug/ml, 24 h). **H.** Proliferation of HepG2 cells in presence of M2 macrophage conditioned media or unconditioned controls, and TGF-β or inhibitor LY2157299 were added as indicated. **I.** Migration and invasion of HepG2 cells in presence of M2 macrophage conditioned media or unconditioned controls, and TGF-β or inhibitor LY2157299 were added as indicated. **J.** Western blot of TGF-β expression in HepG2 derived from experiment. **K.** Western blot of TGF-β expression in HepG2 of 24 h serum-derived HepG2 cultures, treated with sorafenib (5 ug/ml, 3 h) followed by stimulation with TGF-β (100 ng/ml, 3 h) as indicated.

We measured HepG2 and Huh7 cells growth in the presence of media derived from sorafenib pretreated M1 and M2 macrophage cultures in a time course experiment to understand the effect of sorafenib treatment on growth factor release by macrophages. Macrophage medium was exchanged after sorafenib treatment to avoid direct inhibitory effects on hepatoma cells. Pretreatment with sorafenib (5 μg/ml) reduced M2-induced Huh7 and HepG2 cells proliferation during the 8 days period. Thus, sorafenib had a dose-dependent anti-proliferative effect. Inhibition of the growth promotion reached 47.0 + 12.0% (mean + SD) and 64.0 + 5.5% (mean + SD). In contrast, no relevant effects on M1 macrophage-driven growth induction were observed (Figure 5A–5D). Sorafenib in M2 supernatant showed a significant decrease in the number of invading cells compared with the control in HepG2 and Huh7 cells (Figure 5E). Sorafenib upregulated the epithelial marker E-cadherin and downregulated the mesenchymal markers vimentin and fibronectin (Figure 5F).

We performed supplementation experiments to determine which growth factor blocked by sorafenib was essential for M2 macrophage-promoted hepatoma cell growth. Supplementation of TGF-β to the medium of sorafenib-treated M2 macrophage cultures restored HepG2 and Huh7 cells proliferation (Figure 5G). To confirm the contribution of M2 macrophage-derived TGF-β signaling on hepatoma cell growth, we performed TGF-β receptor blocking experiments using the specific phosphorylation inhibitor LY2157299. As expected, TGF-β and M2 macrophage culture supernatant induced HepG2 cells growth and TGF-β phosphorylation. Most importantly, inhibition of TGF-β receptor phosphorylation reduced HepG2 cells proliferation in a dose-dependent manner (Figure 5H). LY2157299 elicited its effect at low concentrations, minimizing potential off-target activity. From these experiments, we conclude that sorafenib inhibits macrophage release of TGF-β, a pivotal growth factor for macrophage-driven hepatoma cell growth and metastases (Figure 5I–5K).

### Effects of Sorafenib on macrophage cells *in vivo*

**Figure 6:**
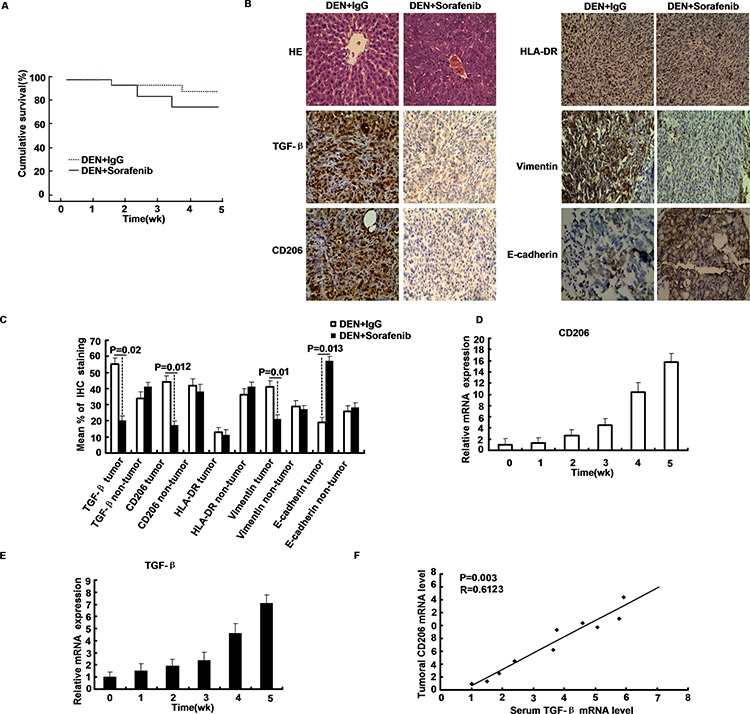
Distribution of macrophages in HCC and macrophage activity during sorafenib treatment **A.** Sorafenib treatments significantly improved survival in the livers of mice administrated with DEN. **B–C.** TGF-β, CD206, HLA-DR, vimentin and E-cadherin staining of sorafenib and IgG treated mice livers. **D–E.** The mRNA levels of TGF-β, CD206 in the liver tissue obtained from mice administrated with DEN at the indicated time intervals. **F.** The relation between the mRNA levels of TGF-β and that of CD206 was assessed by Pearson's correlation test.

To explore the role of sorafenib in hepatocarcinogenesis, we examined the status in the livers of mice administrated with DEN. Treatment with sorafenib as monotherapy significantly decreased HCC burden compared to IgG controls (*p* < 0.01) (Table 1). Treatment with sorafenib improved survival (*p* < 0.05) (Figure 6A). The M2 marker CD206 was significantly decreased in the tumor tissue (*p* = 0.012), and down-regulation of TGF-β occurred in HCC tissue treated with sorafenib (*p* = 0.02). A significant down-regulation of vimentin and up-regulation of E-cadherin were seen in HCC tissue treated with sorafenib (*p* < 0.05) (Figure 6B–6C). However, the M1 marker HLA-DR showed no difference; CD206 levels in the non-tumorous tissue were unaltered after treatment with sorafenib.

**Table 1:** Dysplastic and HCC lesions of treatment with sorafenib or IgG

		IgG	Sorafenib	*P* value
HCC (*n* = 10)	Mean	2.78	1.85	0.031
	SEM	0.45	0.32	
Size(μm^2^)	Mean	1485654.4	513421.6	0.0011
	SEM	418161.3	51112.7	
HCC burden	Mean	5368187.9	1345157.6	0.00034
	SEM	1896100.8	342868.5	

HCC burden is calculated as size × number.

Developed HCC with the up-regulation of TGF-β in liver serum, which implied the essential role of TGF-β in HCC. Notably, expression of M2 macrophage marker which includes CD206 was also increased during mice hepatocarcinogenesis and closely correlated with the up-regulation of TGF-β (R = 0.6123, *p* = 0.003) (Figure 6D–6F). The M2 marker CD206 was significantly decreased in surrounding and tumor tissues of Sorefenib-treated mice, compared to IgG controls (*p* < 0.05). The M1 marker HLA-DR was slightly decreased in sorafenib-treated tumors (Table 2). More importantly, a small portion of macrophage in DEN-treated mice cirrhotic livers were found to co-express CD206, indicating that the macrophage may acquire tumor initiating cell features during carcinogenesis. These results suggest the importance of TGF-β in the generation of macrophage during hepatocarcinogenesis.

**Table 2:** CD206 and HLA-DR expression of Sorefenib-treated

			DEN + IgG	DEN + Sorafenib	*P* value
Tumor	CD206	Mean	1.92	0.53	0.001
		SEM	0.54	0.19	
	HLA-DR	Mean	1.56	0.49	0.07
		SEM	0.56	0.31	
Non-tumor	CD206	Mean	0.92	0.62	0.521
		SEM	0.28	0.21	
	HLA-DR	Mean	0.79	0.38	0.62
		SEM	0.34	0.29	

### Macrophage and secreted TGF-β clinically correlated with HCC

**Figure 7:**
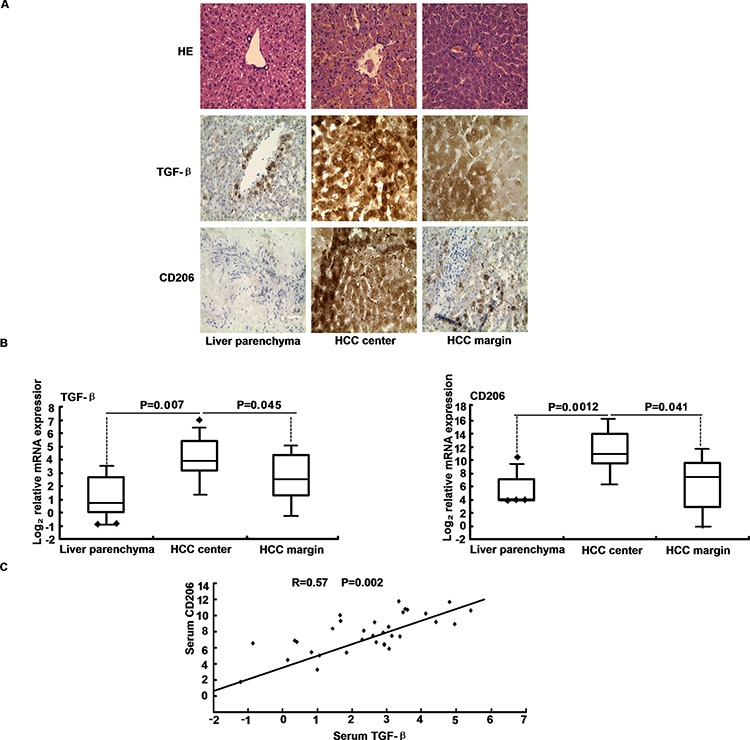
TGF-β levels correlate with macrophage marker expression in HCC **A.** TGF-β and CD206 protein expression were determined in HCC compared to liver parenchyma by immunohistochemical. **B.** The HCC patients and control hepatic tissue were subjected to qRT-PCR analysis of TGF-β and CD206. **C.** Serum TGF-β and CD206 mRNA expression levels positively correlated in HCC samples. Statistical analysis to evaluate correlation was performed using Pearson's correlation analysis.

The presence of M2-polarized TAMs in HCC tissue was measured using CD206 specific stains of curatively respected HCC. Because truncated CD206 is shed into serum by activated macrophages during inflammation, it can serve as a biomarker for M2 macrophage response *in vivo* (Figure 7A). We concluded that M2 cells were activated in HCC patients. As expected, interestingly, CD206 were scarcely expressed in non-tumors but highly expressed in HCC along with up-regulated TGF-β (Figure 7B). Analysis of CD206 and TGF-β expression has referential significance for clinical gene-targeted therapy. Increased CD206 expression in liver cancer indicates a poor prognosis (Table 3). Furthermore, TGF-β levels were positively correlated with CD206 expression in HCC, implying the effect of CD206 and TGF-β on the transformation of HCC (R = 0.57, *p* = 0.002) (Figure 7C). Considering the consistent results of DEN-induced mice hepatocarcinogenesis, we speculated that the macrophage cells may undergo malignant transformation towards HCC, where the unique TGF-β exposure may play an important role.

**Table 3:** Clinicopathological features and Serum CD206 expression in HCC

Clinicopathological features		Number (*n* = 32)	Serum CD206 (pg/ml)		*p* value
			< 100	≥ 100	
Age	< 40	13	8	5	0.618
	≥ 40	19	10	9	
Gender	male	15	9	6	0.688
	female	17	9	8	
AFP (ug/L)	< 400	15	10	5	0.265
	≥ 400	17	8	9	
ALT (U/L)	< 40	14	4	10	0.005
	≥ 40	18	14	4	
AST (U/L)	< 40	16	6	10	0.033
	≥ 40	16	12	4	
TGF-β(pg/mL)	< 100	12	3	9	0.006
	≥ 100	20	15	5	
Tumor size(cm)	< 5	17	8	9	0.265
	≥ 5	15	10	5	
Cirrhosis	Yes	13	7	6	0.821
	NO	19	11	8	
TNM staging	I	12	3	9	0.02
	II	10	8	2	
	III	10	7	3	

## DISCUSSION

Sorafenib is a multi-kinase inhibitor that suppresses HCC growth and represents the sole approved systemic therapy for HCC. Tyrosine kinase inhibitors are promising candidates for TAM-directed therapy [22] and sorafenib is a standard palliative treatment for HCC [23]. TAMs located in the tumor microenvironment increase HCC progression, TAMs thereby foster tumor cells proliferation and tumor spread. Interestingly, the macrophage M2 phenotype resembled by TAMs changed under sorafenib treatment. Sorafenib treatment reversed alternative macrophage polarization, indicated by a shift of phenotypic or functional markers (IL-6, IL-10 and CD206), towards a classical M1 polarization state. In serum samples from HCC patients, elevated CD206 indicated a M2-type macrophage activation, which, along with CD206 release is correlated with unfavorable clinical outcomes of HCC.

Sorafenib blocks different tyrosine kinases, such as Ras, Raf and ERK, inhibiting proliferation and survival of tumor cells in addition to anti-angiogenic effects, these effects promote HCC regression [24]. In response to the stimulation of pathogen-associated molecular patterns, MAPKs are activated rapidly, leading to the production of proinflammatory cytokines [25]. Therefore, the threshold and magnitude of MAPKs activation must be tightly controlled to modulate inflammatory responses. Sorafenib subverts immune responses by mitigating MAPKs [26].

DUSP1 preferentially dephosphorylates activated p38 and JNK relative to ERK1/2 [27]. In LPS-stimulated macrophages, DUSP1 is transiently expressed and rapidly induced, and it returns to basal levels quickly [28]. It is a critical negative regulator of macrophage signaling in response to inflammatory stimuli and is responsible for switching off the production of proinflammatory cytokines and its expression has been found to correlate with cancer development and progression. Up-regulation of DUSP1 in the early phase of cancer helps the tumor to evade JNK1-induced apoptosis, whereas down-regulation of DUSP1 allows for proliferation and increased tumor mass in the more advanced stages of tumorigenesis [29].

Previously, we reported that miR-101 was frequently down-regulated in HBV-positive HCC tumor tissues compared with adjacent noncancerous tissues, suggesting that miR-101 may play a tumor-suppressive role in HCC development. Here, we found that DUSP1 is a direct target of miR-101 and that stimulation of the LPS-activated PI3K/AKT pathway to induce miR-101 expression and that miR-101 expression repressed DUSP1 expression, prolonging activation of MAPKs. Collectively, our data suggest an essential role for miR-101 in regulating innate immune responses to LPS stimulation. Because miR-101 expression is inhibited by sorafenib, repression of DUSP1 by miR-101 may be a general mechanism by which proinflammatory cytokine production is regulated by LPS. Therefore, sorafenib differential regulation of MAPKs phosphorylation strongly suggests that the upstream regulator may be DUSP1.

PI3K-deficient cells had enhanced p38 activation and IL-12 production [30]. An explanation for this may be that PI3K/AKT suppresses p38 activation through inhibition of upstream regulators, apoptosis signal-regulating kinase 1, and MEK kinase 3 [31, 32]. Our research shows that M2 cells treated with PI3K inhibitor LY294002 inhibits LPS-induced activation of JNK, suggesting PI3K is a positive regulator of MAPKs. We also observed that inhibition of PI3K by LY294002 or specific inhibition of AKT expression by siRNA decreases activation of MAPKs. In addition, inhibition of PI3K enhances DUSP1 expression. Moreover, cells treated with LY294002 markedly attenuated miR-101 expression induced by LPS. Thus, PI3K may function as a positive regulator of innate immune responses, and the discrepancy may be due to different cell types used or different experimental conditions. We observed that PI3K/AKT regulates miR-101 expression, thereby up-regulating DUSP1. Inhibition of PI3K/AKT by PI3K inhibitors suppressed miR-101 induction by LPS, leading to enhanced DUSP1 production and subsequent p38 and JNK inhibition. Thus, PI3K/AKT likely negatively regulates DUSP1 expression through induction of miR-101.

Previously, we noted that TGF-β was an inducer of early hepatic dysplasia and HCC growth, suggesting clinical relevance. TGF-β is the most potent hepatic pro-fibrogenic cytokine produced by activated mesenchymal cells upon chronic liver damage [33] and it is suggested to be a multifunctional cytokine that exerts biological effects on tissue and organ development, cellular proliferation, differentiation, survival, and apoptosis [34]. TGF-β is thought to be a link among chronic injury, cirrhosis, and HCC [32]. Accumulating evidence indicated that TGF-β modulates expression of numerous genes relevant to tumor development [35], so it is thought to have a central role in the EMT, a critical cellular event during tumor metastasis [36]. HCC usually occur in cirrhotic livers in which TGF-β is highly expressed compared with healthy controls, indicating a possible pro-oncogenic role of TGF-β in HCC initiation [35]. If the effect of miR-101 is solely through DUSP1, then TGF-β regulation may be an early observation. However, we found that miR-101 had no such effect on TGF-β secretion in the first 24 h, whereas TGF-β production was substantially higher in cells transfected with miR-101 at 72 h. Therefore, miR-101 also may regulate TGF-β production via targeting expression of other genes. Macrophages are a relevant source of active TGF-β, which can be targeted by sorafenib. We postulate that sorafenib-triggered changes of macrophage polarization are responsible for reduced TGF-β secretion and macrophage-induced HCC growth. Accordingly, complete macrophage marker depletion improved tumor response to sorafenib in HCC harboring mice. In our study serum CD206 which is an indicator of macrophage activation decreased after sorafenib treatment *in vivo* and positively correlated with TGF-β.

**Figure 8:**
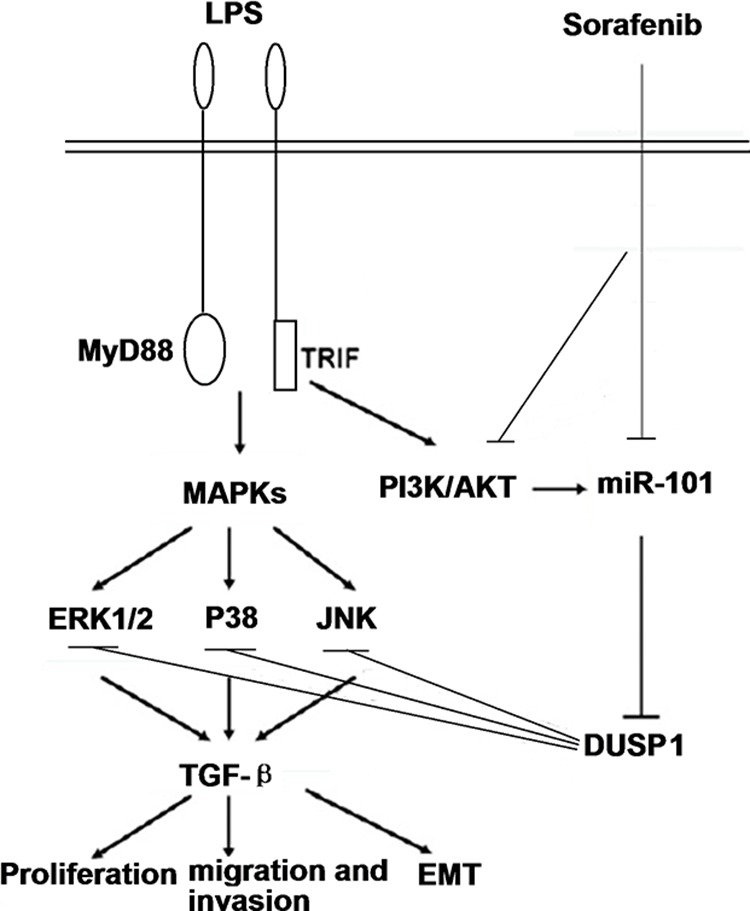
A schematic model depicted the hypothesized molecular mechanism miR-101 targets DUSP1 to regulate the activation of MAPKs in sorafenib inhibits macrophage-induced growth of HCC progression by interference with TGF-β secretion.

In summary, sorafenib alters the function of M2-polarized TAMs and reduces TGF-β-driven HCC progression. miR-101 directly targets DUSP1 to regulate MAPKs activation and subsequent cytokine production in response to LPS stimulation (Figure 8). These data suggest a complexity of signal transduction pathways involved innate immune responses to sorafenib.

## MATERIALS AND METHODS

### Cell culture

CD14+/− monocytes were isolated from PBMC by magnetic bead positive selection (Miltenyi Biotec) and cultured in the presence of 800 U/ml granulocyte-macrophage colony-stimulating factor (GM-CSF) (Bayer Healthcare) for one week to generate M1 macrophages, and 100 ng/ml macrophage colony-stimulating factor (M-CSF) (Peprotech) for one week to generate M2 macrophages. Macrophage medium (RPMI 1640, Gibco) was supplemented with FBS (10%), L-glutamine (1%), penicillin (1%) and streptomycin (1%) (Invitrogen). HepG2 and Huh7 cells were maintained in RPMI 1640 supplemented with 10% FBS. Cells were maintained in a humidified 37°C incubator with an atmosphere of 5% CO2. Transfections were performed with Lipofectamine 2000 kit (Invitrogen) according to the manufacturer's instructions. Double-stranded miR-101 mimics, single-stranded miR-101 inhibitor, or their relative negative control RNA (GenePharma, Shanghai, China) was introduced into cells at a final concentration of 50 nM.

### Reverse transcription and qRT-PCR

RNA was extracted using TRIzol reagent (Invitrogen). Reverse transcription was performed using the M-MLV Reverse Transcription System (Promega). Stem-loop reverse transcription for mature miR-101 and U6 primers was performed as previously described [16]. U6 RNA was used as a miRNA internal control. The primers used for stem loop reverse-transcription PCR for miR-101 were purchased from RIBOBIO (Guangzhou, China). qRT-PCR was performed using a standard SYBR-Green PCR kit protocol on a StepOne Plus system (Applied Biosystems, CA), and β-actin was used as the endogenous control to normalize the relative amount of total mRNA in each sample. The primer sequences are summarized in Table 4. qRT-PCR reactions were performed in triplicate and included no-template controls. Relative expression was calculated using the comparative Ct method.

**Table 4:** Primer sequences for qRT-PCR analysis

	Primer sequence (5′-3′)	
	Forward	Reverse
CD68	GCTACATGGCGGTGGAGTACAA	ATGATGAGAGGCAGCAAGATGG
CD206	TTCGGACACCCATCGGAATTT	CACAAGCGCTGCGTGGAT
HLA-DR	ATCATGACAAAGCGCTCCAACTAT	GATGCCCACCAGACCCACAG
TGF-β	CCCAGCATCTGCAAAGCTC	GTCAATGTACAGCTGCCGCA
DUSP1	GCTGTGCAGCAAACAGTCGA	CGATTAGTCCTCATAAGGTA
β-actin	TCCTGTGGCATCCACGAAACT	GAAGCATTTGCGGTGGACGAT

### ELISA

After stimulation at indicated time points, cell supernatants were collected and analyzed using a Quantikine ELISA Kit (R&D Systems) according to the manufacturer's Instructions.

### Cell proliferation assay

Sorafenib (Santa Cruz Biotechnology, CA) was dissolved in DMSO and was further diluted to 0.01%v/v in cell culture medium to a final concentration of 1.2–5 μg/ml. Mock controls were DMSO (0.01% v/v). After one week of differentiation, sorafenib was added to the cell culture medium of monocyte-derived macrophages for 24 h, followed by a medium exchange and addition of serum-free macrophage medium for 24 h. Cell viability and number was calculated via CCK-8 assay according to instructions provided by the supplier. Substrate turnover after standardized incubation (3 h) was analyzed with a photometer. Serum-free macrophage medium was conditioned in the presence of M1 or M2 macrophage cultures for 24 h. Conditioned medium was transferred to cultured HepG2 and Huh7 cells seeded in a flat-bottom 96 well-plate (1, 000 cells/well) one night before growth kinetic assessment. Conditioned or unconditioned control media was exchanged every 48 h and cell growth was measured by CCK-8 assay. The TGF-βphosphorylation inhibitor LY2157299 (Novartis Pharma) was added to the transferred media in concentrations indicated. Macrophage cultures were stimulated with 1 ng/ml lipopolysaccharide (LPS) (Invitrogen) for 3 h. Recombinant TGF-β (Peprotech) was added in concentrations as indicated.

### Migration and invasion assay

Boyden chamber Matrigel cell invasion assays were performed following the manufacturer's protocol. Briefly, 3 × 10^5^ cells were plated into cell culture inserts with microporous filters (BD, U.S.) coated with (invasion) or without (migration) Matrigel and incubated. After incubation for 48 h, the invaded cells at the bottom of the membrane were stained and counted under a light microscope.

### Luciferase reporter assay

For the luciferase reporter assay, 4 × 10^3^ 293T cells were plated in each well of a 96-well plate. The cells were co-transfected with miR-101 mimics or negative control RNA at a final concentration of 50 nM and 10 ng of either pmiR-RB-REPORT™-DUSP1–3′-UTR-WT or pmiR-RB-REPORT™-DUSP1–3′-UTR-MUT (RIBOBIO, Guangzhou, China) using the calcium phosphate precipitation method. Cells were collected 48 h after transfection and analyzed using the Dual-Luciferase Reporter Assay System (Promega). Relative luciferase activity was normalized to renilla luciferase activity. Transfections were done in duplicate and repeated at least 3 times in independent experiments.

### Western blot

Total cell lysate was prepared in a 1× sodium dodecyl sulfate (SDS) buffer. Equal amounts of protein were separated by SDS-polyacrylamide gel electrophoresis. The proteins were then transferred onto polyvinylidene fluoride membranes. After incubation with antibodies specific for p38, p-p38, ERK, p-ERK, JNK, p-JNK, p-AKT, EMT antibody sampler Kit (Cell Signaling Technology) and DUSP1 (Santa Cruz Biotechnology, CA), the blots were incubated with goat anti-rabbit or anti-mouse secondary antibody (Santa Cruz Biotechnology, CA) and visualized using ECL as previously described [20].

### Immunohistochemistry

Immunohistochemistry for protein expression in HCC tissue was performed using specific antibodies. Briefly, sections were deparaffinized, subjected to microwave antigen retrieval for 15 min in sodiumcitrate solution (pH 6.0), and then incubated with 3% hydrogen peroxide to block endogenous peroxidase activity. The sections were incubated with primary antibody (1:1000 dilution) overnight at 4°C, followed by second antibody (1:100 dilution) at 37°C for one hour. Finally, the sections were counterstained with hematoxylin. Sections stained with PBS only were used as the negative staining control.

### Sorafenib treatment

Mice received DEN (Sigma-Aldrich) to induce HCC after 25 weeks and controls received saline as described [21]. Mice that received DEN for 25 weeks developed HCC and were subsequently treated with 10 mg/kg sorafenib diluted in saline with 20% polysorbate 80 (daily, intragrastric, *n* = 10) and 25 mg/kg IgG (2 × /week, intraperitoneal, *n* = 10) for 5 weeks.

### Clinical specimens

We obtained informed consent from all subjects and the local ethics committee approved the protocol at the first affiliated hospital of Chongqing Medical University P.R. China. All samples were evaluated and histologically evaluated by pathologists. All patients provided informed consent for the study to retain and analyze their tissues for research purposes. The samples were immediately snap-frozen following resection and stored in liquid nitrogen until processing.

### Statistical analysis

Statistical significance of data was determined by the Student's *t* test, and data are expressed as mean ± standard deviation (SD) from at least 3 independent experiments. Pearson correlation analyses were used to examine the correlation of two parameters. Differences were considered statistically significant when *p* < 0.05.
